# Population Pharmacokinetic Analyses for Tebipenem after Oral Administration of Pro-Drug Tebipenem Pivoxil Hydrobromide

**DOI:** 10.1128/aac.01451-22

**Published:** 2023-05-16

**Authors:** H. Ganesan, V. K. Gupta, M. C. Safir, S. M. Bhavnani, A. K. Talley, D. Melnick, C. M. Rubino

**Affiliations:** a Institute for Clinical Pharmacodynamics, Inc., Schenectady, New York, USA; b Spero Therapeutics, Inc., Cambridge, Massachusetts, USA

**Keywords:** tebipenem, population pharmacokinetics

## Abstract

Tebipenem pivoxil hydrobromide (TBP-PI-HBr) is an oral (PO) carbapenem pro-drug that is converted to the active moiety tebipenem in the enterocytes. Tebipenem has activity against multidrug-resistant Gram-negative pathogens, including extended-spectrum beta lactamase-producing Enterobacterales, and is being developed for the treatment of patients with complicated urinary tract infections (cUTI) and acute pyelonephritis (AP). The objectives of these analyses were to develop a population pharmacokinetic (PK) model for tebipenem using data from three phase 1 studies and one phase 3 study and to identify covariates that described the variability in tebipenem PK. Following construction of the base model, a covariate analysis was conducted. The model was then qualified by performing a prediction-corrected visual predictive check and evaluated by using a sampling-importance-resampling procedure. The final population PK data set was composed of data from 746 subjects who provided 3,448 plasma concentrations, including 650 patients (1,985 concentrations) with cUTI/AP. The final population PK model that best described tebipenem PK was found to be a two-compartment model with linear, first-order elimination and two transit compartments to describe the rate of drug absorption after PO administration of TBP-PI-HBr. The relationship between renal clearance (CL_R_) and creatinine clearance (CLcr), the most clinically significant covariate, was described using a sigmoidal Hill-type function. No dose adjustments are warranted on the basis of age, body size, or sex as none of these covariates were associated with substantial differences in tebipenem exposure in patients with cUTI/AP. The resultant population PK model is expected to be appropriate for model-based simulations and assessment of pharmacokinetic-pharmacodynamic relationships for tebipenem.

## INTRODUCTION

Tebipenem pivoxil (TBP-PI) is a carbapenem antibiotic that is a pro-drug of tebipenem, the pharmacologically active moiety. Tebipenem has activity against both Gram-positive and Gram-negative organisms, including multidrug-resistant (MDR) isolates such as extended-spectrum beta-lactamase (ESBL) producers and those resistant to trimethoprim-sulfamethoxazole and fluoroquinolones (1, 2). The hydrobromide salt of TBP-PI, tebipenem pivoxil hydrobromide (TBP-PI-HBr), is currently being developed as an oral (PO) treatment for adults with complicated urinary tract infection (cUTI) including acute pyelonephritis (AP).

Several phase 1 studies had been completed prior to these analyses to characterize pharmacokinetics (PK) of tebipenem. The first of these studies, study SPR994-101 (study 101), determined tebipenem PK to be dose-proportional and linear after single doses of 100 to 900 mg of the immediate release (IR) formulation (3). In the same study, no accumulation was seen over 14 days of administration, and no substantial food effect was observed. A second study, study SPR994-102 (study 102), showed that as renal impairment increased, the tebipenem area under the concentration-time curve (AUC) increased and renal clearance (CL_R_) of tebipenem decreased (4). Study 102 also determined renal impairment to have minimal effect on the maximum plasma concentration (Cmax), and that hemodialysis was effective at removing tebipenem from the blood. The final phase 1 study, study SPR994-104 (study 104), confirmed the dose-dependent exposure observed in study 101 and demonstrated that tebipenem did not produce any clinically significant effects on heart rate or electrocardiogram parameters (5).

Following the completion of these studies, PK data from a phase 3 study in patients with cUTI/AP (study SPR994-301 [ADAPT-PO]) (6) was also available. At this time, a comprehensive population PK model analyses for tebipenem was undertaken. The primary objectives of these analyses were to develop a population PK model to describe the disposition of tebipenem using data from the above-described phase 1 studies and a phase 3 study, and to identify covariates associated with variability in tebipenem PK.

## RESULTS

### Pharmacokinetic analysis population.

For the development of the population PK model, a total of 5,672 tebipenem concentration records from 845 subjects were available from the four studies (study 101, study 102, study 104, and ADAPT-PO). The number of subjects/patients considered for the population PK analyses by study is presented in Table S1 in the supplemental material. All subjects or patients included in the analysis population received the same TBP-PI-HBr IR formulation. The final data set included 3,448 plasma concentrations from 99 phase 1 subjects and 647 phase 3 patients and urine concentrations from 67 phase 1 subjects and 37 phase 3 patients. Tebipenem plasma concentration values identified as outliers were minimal (<3% of phase 3 samples and none from phase 1 subjects). Similarly, no urine samples from phase 3 patients and <5% of urine samples from phase 1 subjects were assessed as outlier concentration values and excluded from the analysis. Approximately 12% of the plasma concentrations from subjects who received the TBP-PI-HBr IR formulation were reported as below the limit of quantitation (BLQ), with 97% of these coming from subjects enrolled in the phase 1 studies. Approximately 85% of these BLQ samples were drawn either within 15 min of a single dose (i.e., during the lag time in absorption) or more than 8 h after a dose.

Demographics for the final PK analysis population, presented by study and overall, are provided in Table 1. In general, patients enrolled in the renal impairment study (study 102) and the phase 3 study (ADAPT-PO) were older (median age of 63.0 and 62.0 years, respectively) than the subjects enrolled in the other two phase 1 studies (median age less than 40 years). Body size covariates (height, weight, body surface area [BSA]) were similar across studies. Median body mass index (BMI) was also similar across studies, with the exception of subjects from study 101, who had lower BMI overall. As expected, the median creatinine clearance (CLcr), as calculated by the Cockcroft-Gault equation (7) and normalized to BSA, was highest in subjects enrolled in studies 101 and 104 and lowest in study 102. The lower median CLcr in the ADAPT-PO study, relative to healthy subjects with normal renal function from studies 101 and 104 is likely a result of (i) the inclusion of patients with CLcr as low as 30 mL/min, and (ii) age-related changes in renal function in the older population enrolled in the study. Overall, the population was 51.3% female. Of note, the study population was predominantly white (95.9% overall and 98.6% in the ADAPT-PO study).

**TABLE 1:** Summary statistics or counts of the baseline subject demographic characteristics of the final PK analysis population^*a*^

Variable	Median (Min to Max) or *n*/*N* (%)				
	Phase 1			Phase 3	Pooled dataset (*n* = 746)
	Study 101 (*n* = 36)	Study 102 (*n* = 39)	Study 104 (*n* = 24)	ADAPT-PO (*n* = 647)	
Age (yr)	24.0 (18.0 to 42.0)	63.0 (42.0 to 80.0)	39.0 (19.0 to 63.0)	62.0 (18.0 to 91.0)	60.0 (18.0 to 91.0)
ht (cm)	176 (164 to 196)	170 (154 to 184)	164 (142 to 178)	168 (110 to 202)	169 (110 to 202)
wt (kg)	75.7 (60.1 to 94.8)	83.3 (54.6 to 121)	72.8 (55.0 to 98.9)	75.7 (42.0 to 142)	76.0 (42.0 to 142)
BSA (m^2^)	1.90 (1.65 to 2.21)	1.96 (1.55 to 2.39)	1.78 (1.49 to 2.17)	1.86 (1.32 to 2.70)	1.86 (1.32 to 2.70)
BMI (kg/m^2^)	24.1 (19.2 to 29.3)	28.5 (21.6 to 39.7)	28.2 (22.6 to 32.1)	26.4 (15.3 to 57.9)	26.3 (15.3 to 57.9)
CLcr (mL/min/1.73 m^2^)^*b*^	120 (83.4 to 161)	42.2 (6.90 to 106)	103 (68.3 to 144)	72.2 (19.8 to 192)	74.2 (6.90 to 192)
Sex					
Male	36 (100)	22 (56.4)	6 (25.0)	301 (46.3)	365 (48.7)
Female		17 (43.6)	18 (75.0)	349 (53.7)	384 (51.3)
Race					
White	29 (80.6)	24 (61.5)	24 (100)	641 (98.6)	718 (95.9)
Black		15 (38.5)		6 (0.92)	21 (2.80)
Asian	6 (16.7)			3 (0.46)	9 (1.20)
Other	1 (2.8)				1 (0.13)

a BMI, body mass index; BSA, body surface area; CLcr, creatinine clearance; cm, centimeters; kg, kilograms; m, meter; Max, maximum; Min, minimum; min, minute; mL, milliliter; n or N, number of subjects or observations; yr, years.

b Subject CLcr was calculated from baseline serum creatinine, age, sex, and body weight using the Cockcroft and Gault equation (7) and then normalized to body surface area.

**FIG 1:**
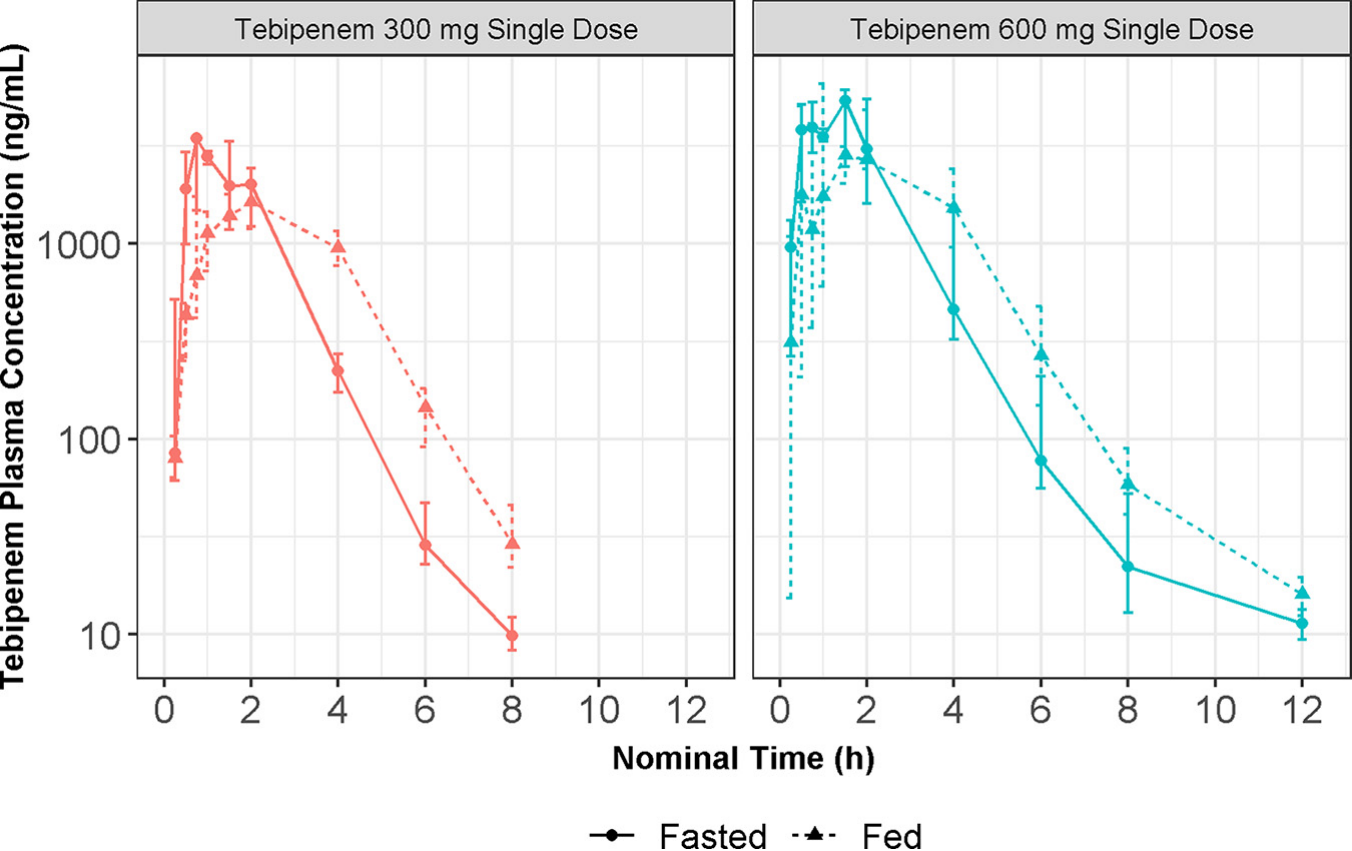
Semilog plots of median (25th to 75th) plasma concentrations versus time, by fed status and dose in healthy subjects (study 101, single-dose, food-effect cohorts). H, hours; mg, milligrams; mL, milliliters; ng, nanograms.

Plots of tebipenem plasma concentration versus time after administration of TBP-PI-HBr 600 mg single oral doses to phase 1 subjects and patients in the phase 3 ADAPT-PO study are presented in Fig. S1 and S2, respectively. The plots of tebipenem concentrations versus time demonstrated that absorption profiles had substantial variability, and there was negligible accumulation at steady state. Plots of the food effect cohorts from study 101 suggested that the presence of food slowed the rate of tebipenem absorption but did not substantially impact overall exposure (Fig. 1).

### Population PK model development.

During base structural model development, models were attempted in which the lag time was replaced with a chain of transit compartments to control the rate of absorption. This was done in anticipation of potential convergence issues when using a lag time to fit the sparse data from the phase 3 study. The transit compartment model provided a comparable fit to that obtained with a lag time model. Thus, the transit compartment model was selected for further model development. Models with either two or three transit compartments were attempted. The model with two transit compartments provided a robust fit with the fewest possible number of transit compartments. It was also necessary to incorporate interoccasion variability (IOV) in the absorption rate constant (Ka) to obtain reasonable fits to the observed data from subjects from study 101 enrolled in the multiple ascending dose (MAD) cohorts. Once an adequate fit to the plasma concentrations was obtained, modifications were made to the model to allow for fitting of the urine concentrations through estimation of CL_R_, which was defined using a sigmoidal-E_max_ relationship between CLcr and CL_R_.

The phase 1 model was then fit to the pooled phase 1 and phase 3 data. As normalized prediction distribution error (NPDE) and prediction-corrected visual predictive check (PC-VPC) plots revealed substantial bias, further attempts to improve the model were made. These included the exclusion of a relatively small number of influential outliers (described above; <3% of concentrations from phase 3 patients), the addition of a power relationship between CLcr and nonrenal clearance (CL_NR_) to account for differences in CL_NR_ seen in subjects with renal impairment, and the inclusion of an effect of dose on Ka (applied to data from study 104 only). The modified model was then used as the reference model for the covariate analysis. Ultimately, eight covariate-parameter relationships were added into the model as part of the forward selection process:

- Ka (fed status and dose were in base model): BMI and infection status
- Apparent oral clearance (CL/F; CLcr was in base model): BSA
- Apparent oral volume of the central compartment (Vc/F): height and infection status
- Apparent oral distributional clearance (CLd/F): none (due to lack of interindividual variability [IIV] on CLd/F)
- Apparent oral volume of distribution of the peripheral compartment (Vp/F): age, BSA, and infection status

Based upon the evaluation of the full multivariable model, two modifications were implemented to simplify the model without impacting the overall fit: (i) the IIV term for Vp/F was removed due to excessive shrinkage, and (ii) removal of the additive portion of the residual variability (RV) model for plasma. All of the covariate relationships identified during forward selection were retained after backward elimination. Evaluation of resultant model, including the PC-VPC plots and sampling-importance resampling (SIR) analysis, indicated that the following changes were warranted: the magnitude of the effect of age on Vp/F was minimal (0.00224 unit in Vp/F change per year of age) and this term was removed from the model; separate IIV terms for CL/F were invoked for healthy subjects and infected patients, which resulted in a substantial improvement in the PC-VPC plots; and, the relationship between BMI and Ka was removed as the SIR indicated that the “true” value for the parameter describing the relationship was not significantly different from 0.

The final population PK model that best described tebipenem PK in both phase 1 subjects and phase 3 patients was found to be a two-compartment model with linear, first-order elimination and two transit compartments to describe the rate of tebipenem absorption after PO administration of TBP-PI-HBr (Fig. S3). The linear elimination was modeled using separate renal and nonrenal clearance terms. This model provided sufficient flexibility in the absorption rate to capture the variability seen in the absorption profiles.

The population PK parameter estimates and associated standard errors for the final population PK model are provided in Table 2 along with the resampled statistics from the SIR analysis. The resampled parameter means are aligned with those estimated in the final model fit, with 90% confidence intervals consistent with the precision of the final model. This suggests that the parameters have been estimated reliably with adequate precision. There is moderate IIV in tebipenem PK as the IIV estimates range from 10.7% (for Vp/F) to 71.9% (for Ka). The shrinkage in the IIV estimates is relatively low with the exception of the IIV on CL/F for phase 1 subjects (66.3%) and Vp/F (77.3%). Overall, the RV in plasma is relatively high (45.7%). However, this is not unexpected given the within-subject variability in the absorption profiles, which manifests as multiple peaks in tebipenem concentrations over the 2 to 4 h after dose administration. Examples of this behavior are provided in the representative plots of individual *post hoc* predicted plasma PK profiles overlaid upon observed data for subjects receiving the 600 mg single dose in phase 1 studies and patients enrolled in the intensive PK cohort of ADAPT-PO (Fig. S4).

**TABLE 2:** Summary statistics of resampled population PK parameters in comparison to the model parameter estimates from the final population pharmacokinetic model^*a*^

Parameter	Final model			Resample statistics (*N* = 1000)^*b*^			
	Estimate	%SEM	Shrink	Mean	Median	%CV	90% CI
CL_NR_	15.6	5.87		15.7	15.7	5.03	[14.4, 17.1]
CL_NR,_ _CLcr power_	0.722	5.92		0.729	0.729	6.09	[0.655, 0.802]
CL_R, MAX_	21.1	5.96		21.1	21	6.67	[18.9, 23.5]
CL_R, CLcr 50_	44.7	7.06		44.8	44.6	8.8	[38.9, 51.5]
CL_R, Hill_	2.13	5.13		2.13	2.13	6.04	[1.92, 2.34]
CL/F:BSA (slope)	0.479	21		0.47	0.471	20.5	[0.311, 0.625]
Vc/F	38.5	3.81		38.5	38.4	4.25	[35.8, 41.3]
Vc/F:Infection status	−0.29	20.4		−0.305	−0.307	−18.5	[−0.396, −0.207]
Vc/F:HTCM (power)	2.09	24.7		2.11	2.11	24.5	[1.26, 2.99]
CLd/F	2.23	11		2.33	2.3	15.2	[1.82, 3]
Vp/F	4.84	6.6		4.93	4.9	8	[4.3, 5.61]
Vp/F:BSA (slope)	0.491	21.3		0.481	0.48	22	[0.306, 0.661]
Vp/F:Infection status	−0.245	19.7		−0.235	−0.235	−21	[−0.318, −0.153]
Ka (fasted)	1.23	5.08		1.24	1.24	7.25	[1.1, 1.39]
Ka (fed)	3.04	4.21		3.06	3.05	4.83	[2.84, 3.3]
Ka:Dose effect	−0.478	3.45		−0.474	−0.475	−6.28	[−0.522, −0.424]
Ka:Infection status	0.368	39.4		0.365	0.364	32.7	[0.168, 0.558]
IIV_CL_ (phase 1)	0.0614 (24.8 %CV)	14.8	66.3	0.0633	0.0617	17.7	[0.0474, 0.0827]
IIV_CL_ (phase 3)	0.328 (57.2 %CV)	5.24	13.3	0.324	0.322	7.37	[0.287, 0.366]
IIV_Vc/F_	0.197 (44.4 %CV)	10.6	41.5	0.205	0.204	15.6	[0.157, 0.26]
IIV_Vp/F_	0.0115 (10.7 %CV)	46.4	77.3	0.0123	0.0114	45.2	[0.00458, 0.0223]
IIV_Ka_	0.518 (71.9 %CV)	8.46	24.0	0.529	0.528	10.4	[0.44, 0.617]
IOV_Ka_ (Occ. 1)	0.201 (44.8 %CV)	37.8	90.2	0.217	0.2	42.9	[0.102, 0.397]
IOV_Ka_ (Occ. 2)	0.201 (44.8 %CV)		92.0				
RV_prop, plasma_	0.209 (45.7 %CV)	2.79	13.9	0.209	0.209	3.47	[0.198, 0.221]
RV_prop, urine_	0.298 (54.6 %CV)	11.3	11.3	0.303	0.302	10.3	[0.254, 0.355]
RV_add, urine_	482 (21.9 ng/mL)	16.4	16.4	508	499	26.2	[308, 752]

a BSA, body surface area; CLcr, creatinine clearance; CLd/F, apparent oral distributional clearance; CL/F, apparent oral clearance; CI, confidence interval; CL_NR_, nonrenal clearance; CL_R_, renal clearance; %CV, percent coefficient of variation; HTCM, height in centimeters; IIV, interindividual variability; IOV, interoccasion variability; Ka, absorption rate constant; N, number of subjects or observations; RV, residual variability; %SEM, percent standard error of the mean; Vc/F, apparent oral volume of the central compartment; Vp/F, apparent oral volume of distribution of the peripheral compartment.

b Resample statistics from the sampling-importance-resampling (SIR) analysis.

This analysis identified several patient characteristics that are associated with the variability in tebipenem PK. During structural model development, three important relationships were identified: (i) between CLcr and both CL_R_ and CL_NR_, (ii) between fed status and Ka, and (iii) between the dose and Ka in study 104 (where subjects received two doses of tebipenem in crossover fashion). Step-wise covariate model building also identified several statistically significant relationships between body size (BSA or height) and/or infection status and the IIV in PK parameters. For body size, BSA was associated with the IIV in CL/F and Vp/F while height was associated with the IIV in Vc/F. Infection status was associated with the IIV in Vc/F, Vp/F, and Ka. The equations describing the covariate relationships (relative to the population mean PK parameters) are provided in equations 1 to 6 below:

(1) $$CL_{\mathrm{NR}}\;=10.2\times {\left[\frac{\mathrm{CLcr}}{\mathrm{79}\mathrm{.32}}\right]}^{0\mathrm{.722}}$$

(2) $$CL_{R}\;=21.1\times \frac{\mathrm{CLc}r^{2\mathrm{.13}}}{\left(\mathrm{CLc}r^{2\mathrm{.13}}\;+\;44.7^{2\mathrm{.13}}\right)}$$

(3) $$\mathrm{CL/F}=\left(CL_{\mathrm{NR}}\;+\;CL_{R}\right)\times \left[1\;+\;\mathrm{0.479}\times \left(\mathrm{BSA-1}\mathrm{.86}\right)\right]$$

(4) $$\mathrm{Vc/F = 38}\mathrm{.5}\times {\left(\frac{\mathrm{HTCM}}{\mathrm{169}}\right)}^{2\mathrm{.09}}\times \left[\mathrm{1- 0}\mathrm{.290}\times \left(\mathrm{1-Infected}\right)\right]$$

(5) Vp/F = 4.84×[1+0.491×(BSA-1.86)]×[1- 0.245×(1-Infected)]

(6) $$\begin{array}{l} \mathrm{Ka =}\left[\mathrm{3.04}\times \mathrm{FAST+1}\mathrm{.23}\left(\mathrm{1-FAST}\right)\right]\times 1\;+\;\mathrm{0.368}\times \left(\mathrm{1-Infected}\right)\times \\ \left[\mathrm{1-0}\mathrm{.478}\times \left(\frac{\mathrm{DOSEMG}}{\mathrm{1200}}\right)\times \mathrm{S104}\right] \end{array}$$

where S104 is a flag variable to indicate if subject was enrolled in study 104.

**FIG 2:**
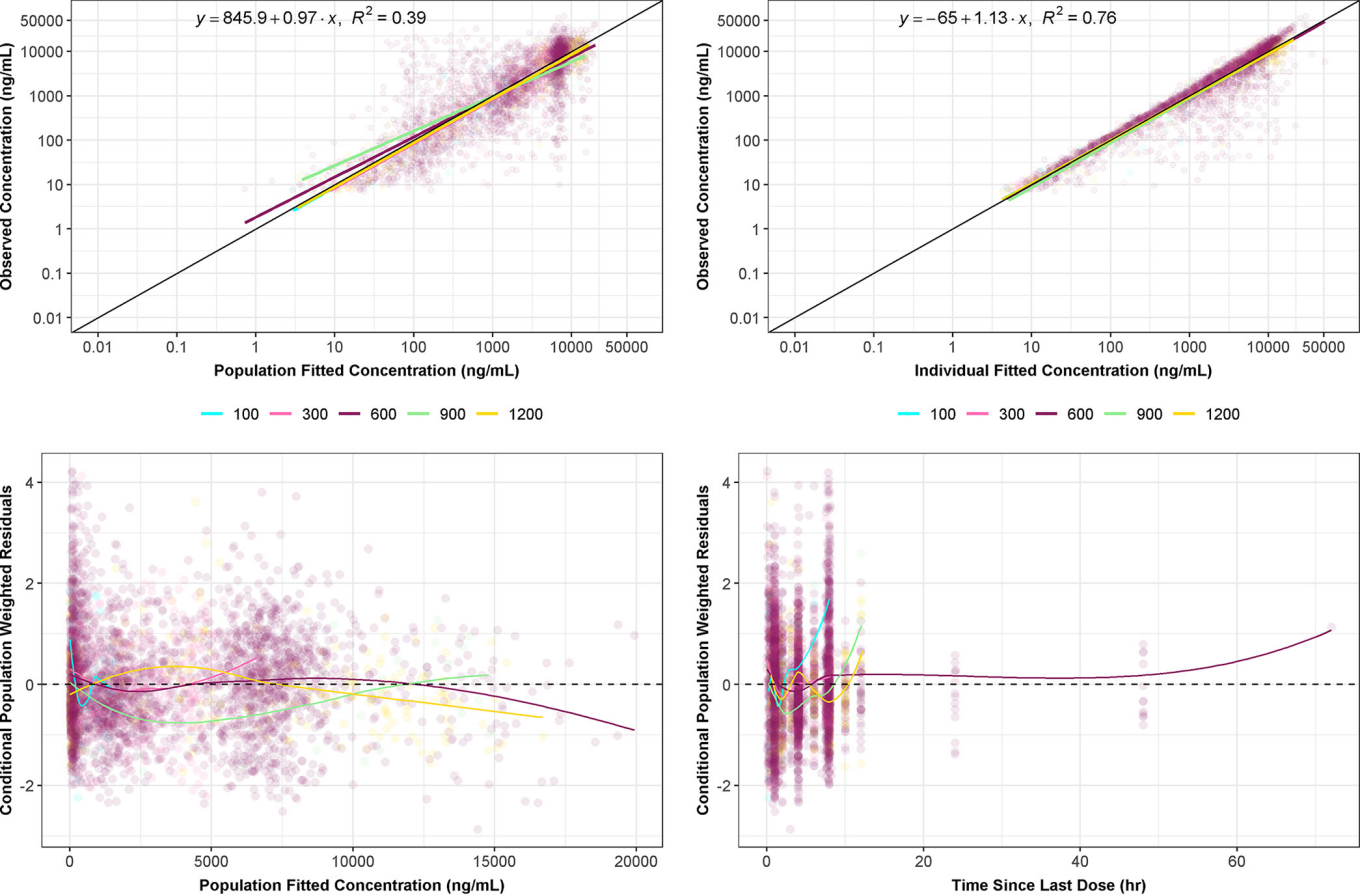
Standard goodness-of-fit plots for the final population PK model (plasma concentrations), colored by dose. hr, hours; mL, milliliters; ng, nanograms. Dose levels are in milligrams (mg).

The goodness-of-fit plots for the final model are provided in Fig. 2 for plasma concentrations. These plots demonstrate the adequacy of the model fit across healthy subjects and infected patients, with an overall coefficient of determination (r^2^) of 0.76 based on observed versus individual predicted plasma concentrations. The line of best fit indicates that the model fit the data with very little bias as the slopes are approximately one and the intercept term for the individual predicted plot is close to zero. The goodness-of-fit plots for the final model for urine concentrations are provided in Fig. S5.

**FIG 3:**
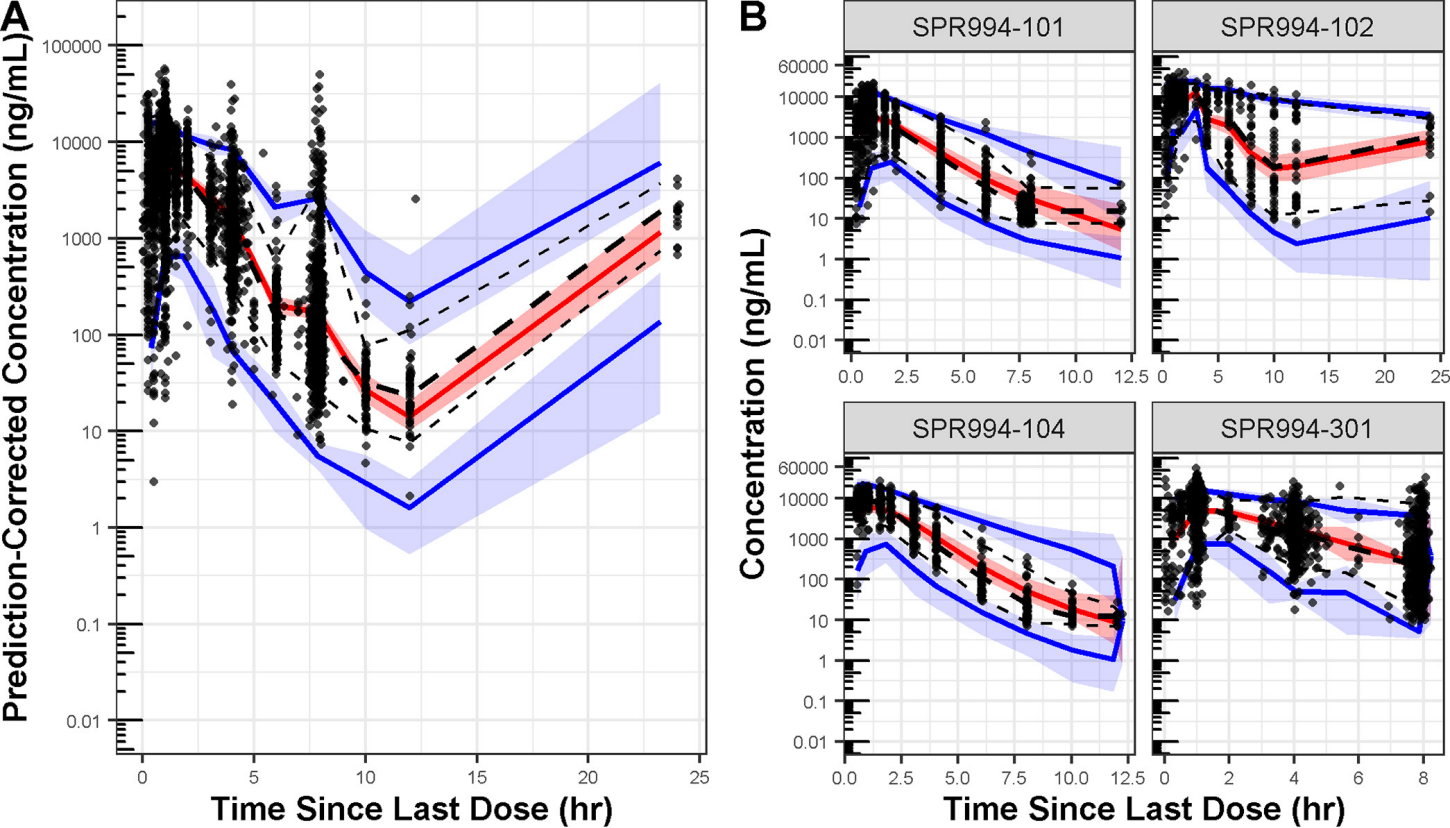
Prediction-corrected visual predictive check plot for the final model using the pooled analysis data set (panel A) and by study (panel B). hr, hours; mL, milliliters; ng, nanograms. Circles represent prediction-corrected observed plasma concentrations while the black lines represent the median (thicker dashed line) and 5th and 95th percentiles (thin dashed lines) of the observed data. The red shaded region shows the 90% prediction interval for the median simulated values and the solid red line is the median of the median simulated values. The blue shaded regions show the 90% prediction intervals for the 5th and 95th percentiles of the simulated values and the solid blue lines show the median of the 5th and 95th percentiles of the simulated values.

The PC-VPC plots (Fig. 3) showed good agreement between median simulated plasma concentrations based on the final population PK model and the median observed plasma concentrations for the pooled data set and when evaluated by study. Overall, the agreement is not as strong for the variability in the observed concentrations as the 5th and 95th percentiles of the observed data do not universally fall within the confidence intervals of the corresponding simulated values. In general, the model is predicting a higher degree of variability than is seen when pooling the data across studies and calculating the summary statistics of the observed tebipenem concentrations over time (Fig. 3, panel A). However, when evaluated by study (Fig. 3, panel B), the degree of bias is relatively small and acceptable given the observed within-subject variability in drug concentrations over time (Fig. S4) and the intended purpose of model-based simulations from this model (i.e., to simulate tebipenem concentration-time profiles in infected patients).

### Exposures and secondary PK parameter estimates.

Summary statistics for key tebipenem PK exposure parameters are provided in Table 3 for patients in the ADAPT-PO trial and presented by renal function and dosing regimen.

**TABLE 3:** Summary (median [Min. to Max.]) of key tebipenem PK parameters in patients with cUTI/AP from the ADAPT-PO study, derived from the fit of the population PK model, stratified by renal function category and TBP-PI-HBr dosage regimen^*a*^

Parameter	CLcr > 30 to ≤ 50 mL/min 300 mg q8h (*n* = 55)	CLcr > 50 mL/min 600 mg q8h (*n* = 595)
AUC_0−24_ (μg•h/mL)^*b*^	59.2 (11.5 to 273)	60.5 (18.0 to 669)
Cmax (μg/mL)^*c*^	6.68 (1.31 to 18.2)	7.98 (1.41 to 51.2)
CL/F (L/h)	15.7 (1.77 to 78.4)	30.7 (2.39 to 100)
V_SS_/F (L)	36.7 (26.2 to 54.5)	41.8 (11.9 to 104)
t_1/2,α_ (h)	0.967 (0.365 to 1.46)	0.708 (0.265 to 1.57)
t_1/2,β_ (h)	2.10 (1.40 to 12.9)	1.77 (1.25 to 7.88)

a AP, acute pyelonephritis; AUC_0-24_, area under the concentration-time curve from time zero to 24 h; CL/F, apparent oral clearance; Cmax, maximum plasma concentration; cUTI, complicated urinary tract infection; h, hours; L, liters; mg, milligrams; mL, milliliters; μg, micrograms; n, number of subjects or observations; q8h, every 8 hours; t_1/2,α_, half-life of the alpha phase; t_1/2,β_, half-life of the beta phase; V_SS_/F, apparent oral steady-state volume of distribution.

b The arithmetic mean AUC_0-24_ estimates were 73.3 and 74.7 μg•h/mL for the 300 mg q8h and 600 mg q8h dose groups, respectively, and 74.6 μg•h/mL for both groups combined.

c The arithmetic mean Cmax estimates were 7.06 and 8.47 μg/mL for the 300 mg q8h and 600 mg q8h dose groups, respectively, and 8.35 μg/mL for both groups combined.

**FIG 4:**
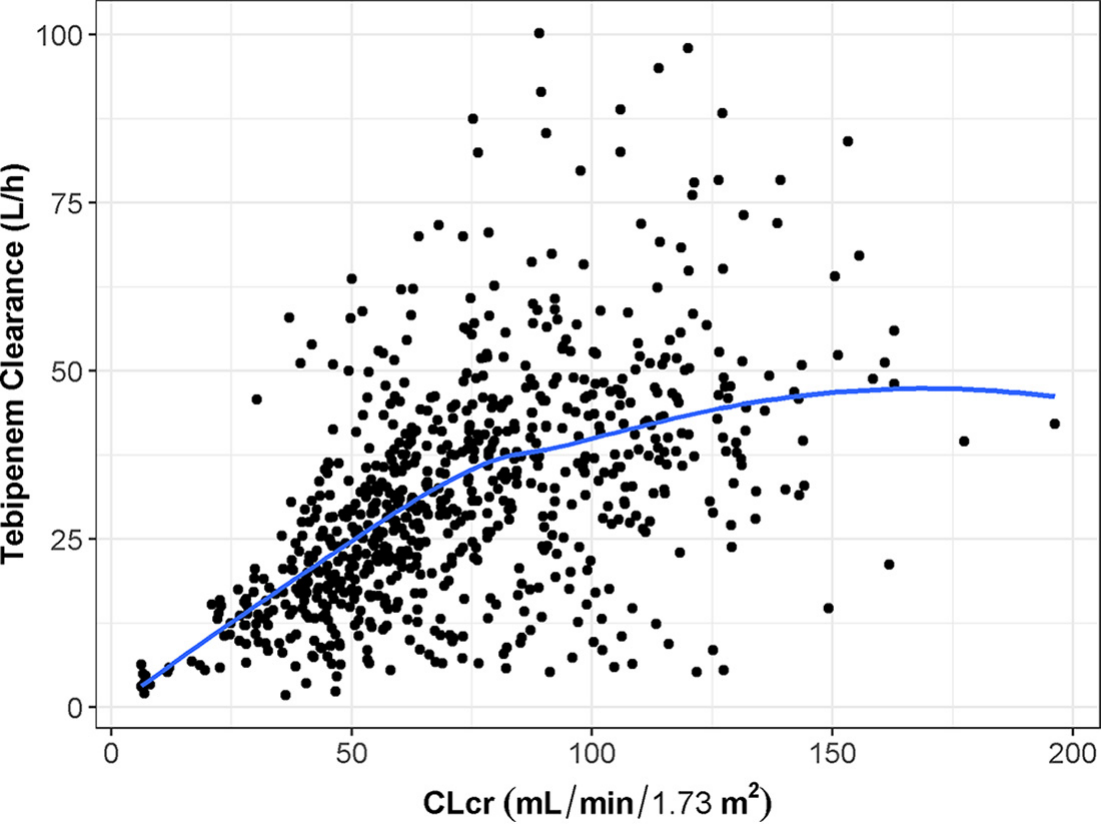
Scatterplot of *post hoc* estimates of tebipenem CL/F versus CLcr. CL/F, apparent oral clearance; CLcr, creatinine clearance; h, hours; L, liters; m, meters; min, minutes; mL, milliliters. The blue line represents the population mean value for CL/F over the range of CLcr values observed in the population PK data set.

During population PK model development, a statistically significant relationship was identified between tebipenem CL/F and renal function, wherein tebipenem clearance increased in a sigmoidal fashion with increasing CLcr. Note that patients who received the 300 mg every 8 h (q8h) regimen of TBP-PI-HBr had baseline CLcr below 50 mL/min/1.73 m^2^; this impact of renal function is reflected in the lower CL/F estimates in Table 3 for those receiving the 300 mg regimen. Fig. 4 also demonstrates this relationship, in which tebipenem clearance increases in an approximately linear fashion up until a CLcr of approximately 80 mL/min/1.73 m^2^. In patients with CLcr ≤ 50 mL/min/1.73 m^2^, the CL/F of tebipenem decreased sufficiently to warrant dose reductions. Box-and-whisker plots showing the distribution of day 1 AUC from time 0 to 24 h (AUC_0-24_) by TBP-PI-HBr dosing regimen in patients from the ADAPT-PO study (Fig. S6) indicated that the dose reduction employed in ADAPT-PO for patients with baseline CLcr below 50 mL/min/1.73 m^2^ is resulting in consistent tebipenem exposure across the study population and supports the appropriateness of the dose reduction algorithm employed in that study.

Older patients tended to have higher tebipenem exposure (AUC_0-24_ and Cmax), slower CL/F, and longer half-life estimates. Overall, the differences between older and younger patients is not of sufficient magnitude to warrant dose adjustments in the elderly other than adjustment required based upon renal function.

Two different measures of body size were identified as significant covariates in relation to various parameters in the population PK model: BSA was significant for CL/F and Vp/F, and height was significant for Vc/F. Scatterplots of the relationships between body weight, BSA, or BMI and tebipenem AUC_0-24_ (data not shown) indicated that there are no trends for AUC_0-24_ to increase with decreasing body size. Therefore, no dose adjustments based upon patient body weight, BSA, or BMI are indicated.

Based on the covariate analyses, sex was not a statistically significant predictor of the IIV in tebipenem PK. There was a high degree of concordance in PK between females and males. The only parameter that appears to be slightly different between the two sexes was the apparent steady-state volume of distribution (V_SS_/F), which was lower in females. This was likely because females tended to have lower height and lower BSA (the two main demographic factors related to the IIV in Vc/F and Vp/F).

## DISCUSSION

The primary objective of these analyses was to develop a population PK model for tebipenem after oral administration of TBP-PI-HBr in healthy subjects, patients with renal impairment, and patients with cUTI/AP. A key facet of the model development was the identification of subject-specific factors associated with the IIV in tebipenem PK. The model was then qualified to demonstrate its robustness to estimate tebipenem exposure in the phase 3 patients and that model-based simulations would reliably predict tebipenem PK in future patients. Finally, the model-predicted exposures from the phase 3 patients were used to explore potential differences in PK among various subgroups.

The data set used to construct the model was large and diverse. A total of 746 subjects provided 3,448 plasma concentrations, including 647 patients (1,985 concentration observations) with cUTI/AP who had been enrolled in the phase 3 study. Urine concentration data were available from 67 phase 1 subjects and 37 phase 3 patients and helped inform the relationship between tebipenem CL/F and renal function. Data obtained after administration of only the IR formulation of TBP-PI-HBr was used for model development. This is because the IR formulation is intended for clinical use and was used in the phase 3 study in patients with cUTI/AP (6).

The final population PK model for tebipenem, which best described the pooled data from phase 1 and 3 studies, was a two-compartment model with linear, first-order elimination and two transit compartments to describe the rate of drug absorption after oral administration of TBP-PI-HBr. The transit compartment model was used as it provided a comparable fit to that obtained with a lag time plus single Ka when fit to the phase 1 data alone while providing the mathematical flexibility required to fit the sparse PK data from phase 3, which would be expected to be noninformative for a lag time and result in issues with model convergence. The relationship between CL_R_ and CLcr, the most clinically significant covariate, was described using a sigmoidal Hill-type function; the total CL/F of tebipenem included an intercept to represent CL_NR_, which was also related to CLcr using an exponential function. It is important to note that this somewhat unconventional covariate relationship was required to achieve an adequate fit to the data across subjects with varying renal function. Fixing CL_NR_ to a single value resulted in bias in the fit to the data from subjects with severe renal impairment, suggesting that physiologic changes secondary to severe renal impairment (e.g., electrolyte disturbances) may be affecting nonrenal mechanisms of tebipenem clearance in addition to the expected impact on CL_R_.

Overall, the final model indicated that there is moderate IIV in tebipenem PK as the IIV estimates range from 10.7% for Vp/F to 71.9% for Ka. Separate IIV terms were estimated for phase 1 subjects and phase 3 patients and showed that IIV for CL/F is substantially higher in infected patients (percent coefficient of variation [%CV] of 57.2% versus 24.8%). Although the variability in CL/F can be a consequence of several factors (e.g., increased variability in the extent of absorption and/or increased variability in the clearance of absorbed drug), the higher IIV in phase 3 patients results in more variability in the predicted tebipenem exposures (AUC_0-24_ and Cmax), which is consistent with the variability seen in observed concentrations from phase 3 patients.

Traditional covariate model building techniques were employed to identify those subject descriptors other than renal function (age, body size, sex, etc.) that were associated with the IIV in tebipenem PK. After pooling of the data across the phase 1 and 3 studies, the resultant data set facilitated the identification of several statistically significant relationships: (i) BSA was significantly related to the IIV in CL/F and Vp/F; (ii) height was significantly related to the IIV in Vc/F; and (iii) infection status was significantly related to the IIV in Ka, Vc/F, and Vp/F. In addition, three factors were found to be related to the rate of tebipenem absorption after administration of TBP-PI-HBr: (i) fed status such that absorption was slower in the fed state, (ii) dose such that absorption rate was slower in subjects from study 104 when they received the 1,200 mg TBP-PI-HBr dose, and (iii) infection status as absorption was faster in infected patients. The effect of food was limited to an impact on the rate of absorption; the extent of absorption is not significantly different in the fed and fasted states. Thus, these analyses would suggest that TBP-PI-HBr can be given without regard for food, consistent with the dosing instructions provided in ADAPT-PO. A formal assessment in a separate food effect phase 1 study demonstrated similar conclusion (8). As noted above, the impact of dose on the absorption rate was limited to subjects from study 104, who had received both the 600 mg and 1,200 mg TBP-PI-HBr dose in a crossover fashion. This effect of dose was not apparent in study 101 where subjects received single doses of TBP-PI-HBr ranging from 100 to 900 mg. This observation suggests that the impact of dose on absorption rate is modest (i.e., the overall variability in the absorption profile of tebipenem masks the impact of dose on absorption rate unless tested in a crossover fashion, which limits variability to a certain extent). Finally, the impact of infection status on absorption rate should be interpreted with caution as the PK sampling scheme in phase 3 patients was relatively uninformative for the estimation of Ka. Given that TBP-PI-HBr will only be indicated for patients, the differences in PK between healthy subjects and infected patients are not relevant clinically. Despite the impact of several covariates upon the absorption process, these covariates impact the rate of absorption and not the extent of absorption. Therefore, no impact would be expected on systemic AUC, which is the relevant measure of tebipenem exposure for efficacy in *in vitro* models (9).

Examination of the impact of the tebipenem exposure in various patient subgroups (i.e., renal function, age, body size, and sex) indicated that the only clinically relevant covariate was renal function. As renal function decreases, tebipenem CL/F falls sufficiently to warrant dose reductions in subjects/patients with CLcr less than 50 mL/min/1.73 m^2^. The results of these analyses support the use of a dose of 300 mg q8h TBP-PI-HBr in subjects with CLcr from 30 to 50 mL/min/1.73 m^2^, consistent with the dosing guidelines employed in ADAPT-PO. An evaluation of appropriate tebipenem dosing regimens for patients with severe renal impairment (i.e., CLcr below 30) was evaluated as part of the PK-PD target attainment analyses conducted to support tebipenem dose selection (data not shown).

Overall, a population PK model was successfully developed for tebipenem and considered robust and reliable for the calculation of individual *post hoc* estimates of exposure. The use of TBP-PI-HBr 300 mg PO q8h is appropriate for patients with moderate to severe renal impairment as it resulted in tebipenem exposures that were similar to those observed in patients with mild renal impairment or normal renal function who received TBP-PI-HBr 600 mg PO q8h. No dose adjustments on the basis of age, body size, or sex are warranted. The resultant population PK model was considered appropriate for model-based simulations and assessment of PK-PD relationships for tebipenem efficacy, which will be reported separately.

## MATERIALS AND METHODS

### Study design.

The population PK model was developed using tebipenem plasma and urine concentration-time data collected from subjects enrolled in three phase 1 studies (studies 101 [3], 102 [4], and 104 [5]) and one phase 3 study (ADAPT-PO) (6). A summary of dosing regimens, sampling strategies, and the number of subjects/patients considered for population PK analyses by study is presented in Table S1 in the supplemental material. With the exception of study 101 where multiple formulations were investigated, all studies utilized the IR tablet formulation. Only data from subjects receiving the IR formulation was used in the population PK analyses.

**(i) Study SPR994-101.** Study 101 (ClinicalTrials.gov registration no. NCT03395249) was a two-part, double-blind, placebo-controlled, phase 1 study of the safety, tolerability, and PK of tebipenem following single and multiple ascending doses of TBP-PI-HBr administered orally in healthy subjects (3). A total of 124 subjects aged 18 to 55 years were enrolled. Study subjects were split into multiple cohorts of eight people; these cohorts were randomized in a 3:1 ratio to TBP-PI-HBr or placebo. There were 17 total cohorts, although cohort 15 did not proceed. The MAD part of the study was only conducted in cohorts 4 and 5. All remaining cohorts comprised the single ascending dose (SAD) portion of the study. Cohorts have been described in detail previously (3).

In the SAD cohorts, subjects received single doses of TBP-PI-HBr ranging from 100 to 900 mg in various formulations. Subjects were administered study drug in either a fasted state, or in both a fed and fasted state separated by a 5-day washout period. Subjects in MAD cohorts 4 and 5 received either 300 or 600 mg of the IR formulation of TBP-PI-HBr q8h for 14 days, with only a single dose administered on day 14. Blood samples for PK analyses in both the SAD and MAD cohorts were collected as described in Table S1. For the SAD cohorts, urine samples were collected on day 1 at predose as well as on days 1 to 2 and days 7 to 8 for the SAD cohorts assessing food effect at 0 to 4, 4 to 8, 8 to 12, and 12 to 24 h after the first dose administration. For the MAD cohorts, urine samples were collected on day 1 at 0 to 4 and 4 to 8 h prior to the second dose and on days 14 to 15 at 0 to 4, 4 to 8, 8 to 12, and 12 to 24 h after start of the last dose.

**(ii) Study SPR994-102.** Study 102 (ClinicalTrials.gov registration no. NCT04178577) was a phase 1, multicenter, open-label study to assess the safety, tolerability, and PK of a single dose of TBP-PI-HBr in adults with varying degrees of renal function (4). A total of 40 patients were enrolled and assigned to one of five cohorts depending on renal function. Cohort 1 consisted of subjects who had an estimated glomerular filtration rate (eGFR) of at least 90 mL/min/1.73 m^2^ as measured by the Modification of Diet in Renal Disease (MDRD) equation (10). Cohort 2 consisted of patients with mild renal impairment, defined as having an eGFR of 60 to < 90 mL/min/1.73 m^2^. Cohort 3 consisted of patients with moderate renal impairment, defined as having an eGFR of 30 to < 60 mL/min/1.73 m^2^. Cohort 4 consisted of patients with severe renal impairment, or those with < 30 mL/min/1.73 m^2^. Cohort 5 consisted of those with End-Stage Renal Disease (ESRD) on hemodialysis.

Patients in cohorts 1 through 4 were administered 600 mg TBP-PI-HBr PO as a single dose. Patients in cohort 5 received 600 mg TBP-PI-HBr once on day 1 and once on day 5. The day 1 dose for patients in cohort 5 was administered approximately 2 h after dialysis; the day 5 dose was given about 1 h prior to dialysis. Data from day 5 of cohort 5 was not utilized in these analyses.

Blood samples for the PK analyses were drawn up to 72 h after dose administration, as described in Table S1. Urine samples were collected predose and from 0 to 4, 4 to 8, 8 to 12, 12 to 24, 24 to 48, and 48 to 72 h after dose administration.

**(iii) Study SPR994-104.** Study 104 (ClinicalTrials.gov registration no. NCT04238195) was a phase 1, randomized, double-blind, placebo- and active-controlled, single dose, four-way crossover thorough QT study that was conducted in healthy adult subjects (5). A total of 24 subjects were enrolled. Over four treatment periods, subjects received a single PO dose of four separate treatments, each separated by at least 7 days. Treatments included 600 or 1,200 mg TBP-PI-HBr, 400 mg moxifloxacin, or placebo. Blood samples for PK analyses were collected in each period up to 24 h after treatment administration, as described in Table S1.

**(iv) Study SPR994-301 (ADAPT-PO).** The ADAPT-PO trial (ClinicalTrials.gov registration no. NCT03788967) was a phase 3, randomized, double-blind, double-dummy, multicenter, prospective study to assess the efficacy, safety, and PK of orally administered TBP-PI-HBr compared to intravenous ertapenem in 1,372 patients with cUTI/AP (6). Patients were randomized and enrolled to one of two treatment arms at a 1:1 ratio receiving TBP-PI-HBr or ertapenem. Patients randomized to TBP-PI-HBr were dosed at 600 mg PO q8h, with dose adjustment allowed for moderate renal insufficiency. Those randomized to ertapenem received 1 g IV every 24 h (q24h). All patients received a matching placebo. Patients were treated with study drug for seven to 10 days, or up to 14 days in patients with bacteremia.

The first 70 patients enrolled (35 patients treated with TBP-PI-HBr) were included in a more intensive PK sampling cohort, in which blood samples were collected at four times on day 2 (day 1 for subjects enrolled under protocol version 1) following an oral dose of study drug. Urine samples for PK evaluations among the first 70 patients enrolled were collected on day 1 over roughly three 8-h aliquots starting on day 1 after the first dose of study drug. For all patients enrolled after the first 70 patients, blood samples were collected at three times following an oral dose of study drug on day 2 (day 1 for subjects enrolled under protocol version 1). Scheduled times of whole blood sample collection are described in Table S1.

### Drug concentration assay.

In all studies, whole blood and urine samples (with the exception of study SPR994-104, which did not collect urine samples) were assayed by a validated liquid chromatography-tandem mass spectrometry (LC-MS/MS) method specific for the determination of TBP-PI (blood) and tebipenem (blood and urine) (11). For the development of the population PK model, only tebipenem blood and urine concentrations were used. Tebipenem whole blood concentrations were converted to plasma by employing a multiplication factor of 3.6 for use in the population PK analyses as described elsewhere (4, 5). The calibration curve had a range of 2.00 to 1000 ng/mL for both blood and urine.

### Handling of outliers and samples assayed as having below the limit of quantitation concentrations.

Specific tebipenem plasma concentrations were to be excluded from the population PK analyses for any of the following reasons: date or time of the previous dose was missing, date or time of sample collection was missing, or drug concentration values were determined to be outliers. An outlier was defined as an aberrant observation that substantially deviated from the rest of the observations within an individual or across all study subjects. Suspected outlier observations were tested and, if justified, excluded based on the procedure described below. Data for each subject were fit with and without the suspected outlier. A plasma concentration value was suspected to be an outlier if the difference between the value of the fitted concentration and the observed concentration was at least three error standard deviations, whereby the trajectory of the PK profile was significantly altered. In the instance when an improvement was seen in the fit of the remaining samples for that subject, then the observation was declared a significant outlier and excluded from the analysis.

BLQ concentration values were flagged in the data set and ignored by the analysis program. In all data presentations (except listings), BLQ concentrations were set to zero.

### Development of the structural population pharmacokinetic model.

The population PK analyses were conducted using NONMEM software Version 7.4 (ICON Development Solutions, Ellicott City, MD) implementing the first-order conditional estimation method with interaction (12). Base structural model construction began with a two-compartment model with linear elimination and drug absorption modeled using a lag time and first-order absorption rate constant fit to the plasma and urine tebipenem data from study 101 (using data for subjects given the immediate release dosage form). IIV was modeled for each PK parameter, where appropriate, using an exponential error model assuming these parameters are log-normally distributed. A combined additive plus proportional error model was initially used to describe residual variability in both plasma and urine. The additive plus proportional residual variability model was reduced to a proportional error model by fixing the additive term to zero if it could be shown that the absolute value of the individual weighted residuals was constant when examined graphically over the range of individual predicted concentrations, or if the additive component was estimated to be negligible. Once an adequate fit to the plasma concentrations was obtained, modifications were made to the model to allow for fitting of the urine amounts through estimation of a CL_R_ parameter.

The data were then pooled with the data from the other two phase 1 studies (studies 102 and 104), and the model was refined as necessary. The phase 1 data were then pooled with data from the phase 3 study and the base structural model was refined further to ensure a robust fit to data from healthy subjects and infected patients. Note that TBPM-PI-HBr doses were included in the population PK data set using the administered dose (i.e., mg of TBPM-PI). The fitted model parameter estimates are therefore inherently conditioned on the mass difference between TBP-PI and tebipenem. The parameters are also conditioned on the percentage of the pro-drug that is converted to tebipenem, which is unknown but expected to be approximately 100% as measurable levels of pro-drug in systemic circulation were not observed in study 101 (data on file).

### Covariate analysis.

After an appropriate base structural model was identified, population PK covariate model development was undertaken using forward selection followed by a backward elimination procedure. The following covariates were tested for their ability to explain the IIV in the PK parameters: age, weight, height, BSA, BMI, CLcr (CL/F only), sex, race, and infection status. The forward selection procedure used a *P*-value of 0.05, while the backward elimination procedure used a *P*-value of 0.001. After forward selection, interindividual variability models were re-evaluated. Pair-wise comparisons of the interindividual specific random effect error terms for each parameter (η) were graphically examined for possible correlations using a scatterplot matrix. If correlations were observed between the η for any pairs of PK parameters (e.g., Pearson’s correlation coefficient with α <0.001), an attempt was made to estimate the corresponding covariance (e.g., off diagonal elements of the variance-covariance matrix) between those parameters in the population PK model. In addition, the distribution of the η for each parameter was examined for irregularities (e.g., skewness, bi modalities, etc.) and alternative interindividual error models or transformations (e.g., Box-Cox) were considered if necessary. After making any potential adjustments to the interindividual variability models or the variance-covariance matrix structure, the focus was shifted toward correcting any potential biases or looking for ways to simplify the residual variability model. If necessary, the additive plus proportional residual error model was simplified to a proportional error model at this stage of the analysis if the data did not continue to support the estimation of the more complex model. The overall distribution of the NPDE provided by NONMEM was also evaluated to determine if the residual variability was biased. In addition, if it appeared that the magnitude or the shape of the distribution of NPDE differed when examined stratified by infection status (healthy subjects versus cUTI/AP patients), residual variability was evaluated separately for each population as appropriate. If more than two covariates were included in the full multivariable model, backwards elimination was employed to ensure that all relationships met a more-strict threshold for statistical significance. After elimination of any nonsignificant covariate relationships, the model was again evaluated for potential revisions such as the removal of extraneous covariate relationships (e.g., where the magnitude of the covariate effect was trivial) or modifications to the IIV and RV models.

### Final model evaluation.

The model was qualified by performing a PC-VPC, which graphically examines the agreement between the 5th, 50th, and 95th percentiles of the observed and the individual simulated concentrations across time intervals (13). In addition, the precision of the PK parameters for the final model was evaluated using an SIR procedure.

### Calculation of secondary PK parameters and exposure estimates.

Individual predicted concentration-time profiles for each patient were generated from the Bayesian PK parameter estimates obtained from the final population PK model. This was accomplished by transitioning the NONMEM model code to C++ code so that simulations could be conducted using mrgsolve, a package for R that facilitates simulations from differential equation-based models (14). The Cmax of tebipenem was determined by direct observation using the individual predicted concentrations. The tebipenem AUC_0-24_ was calculated using the linear trapezoidal rule on the individual predicted concentration-time data. Secondary PK parameters were generated directly from the individual *post hoc* PK parameters. V_SS_/F was calculated by summing the *post hoc* volume of distribution estimates. To account for a multiphase disposition, alpha-, beta-, and (if necessary) gamma-elimination half-lives were derived by the method of Gibaldi and Perrier (15).
