# Supplementary material for: Population Pharmacokinetic Analyses for Tebipenem after Oral Administration of Pro-Drug Tebipenem Pivoxil Hydrobromide
Source: Antimicrob Agents Chemother. 2023 May 16;67(6):e01451-22. doi: 10.1128/aac.01451-22 (PMC10269146; doi:10.1128/aac.01451-22)
Supplement: Supplemental file 1 — Supplemental material. Download aac.01451-22-s0001.pdf, PDF file, 1.6 MB [file aac.01451-22-s0001.pdf]

## Supplementary Material

**Table S1**. Description of Phase 1 and 3 TBP-PI-HBr studies included in the population PK analyses

| Study | Phase | Title | Subjects/Patients (n)[a] | TBP-PI-HBr dosing regimens[b] | Scheduled PK sample collection times |
|---|---|---|---|---|---|
| Study SPR994-101 (Study 101, NCT03395249) [1] | 1 | A Two-part, Double-blind, Placebo-controlled, Phase I Study of the Safety, Tolerability and Pharmacokinetics of SPR994 Following Single and Multiple Ascending Doses of SPR994 Administered Orally in Healthy Volunteers | 103 | SAD: 100, 300, 600, 900 mg PO single dose<br><br>MAD: 300 or 600 mg PO q8h for 14 days | SAD: Day 1[c] pre-dose and at 15, 30, 45 minutes and 1, 1.5, 2, 4, 6, 8, 12, 24, and 48 hours<br><br>MAD: Day 1 pre-dose and at 15, 30, 45 minutes and 1, 1.5, 2, 4, 6, and 8 hours. Pre-dose samples also collected on Days 2, 3, 5, 7, 9, 11, and 13. Additional samples collected at the following times relative to the last dose: pre-dose and at 15, 30, 45 minutes and 1, 1.5, 2, 4, 6, 8, 12, 24, 36, and 48 hours |
| Study SPR994-102 (Study 102, NCT04178577) [2] | 1 | A Phase 1, Open-Label Study to Assess the Pharmacokinetics and Safety of Orally Administered Tebipenem Pivoxil Hydrobromide (TBPM-PI-HBr) in Subjects with Various Degrees of Renal Function | 39 | Cohorts 1-4: 600 mg PO single dose<br><br>Cohort 5: 600 mg PO on Day 1 2h after dialysis and Day 5 1h before dialysis | Cohorts 1-4: pre-dose and at 0.25, 0.5, 1, 1.5, 2, 4, 6, 8, 10, 12, 24, 48, and 72 hours<br><br>Cohort 5: Days 1 and 5 pre-dose and at 0.25, 0.5, 1, 1.5, 2, 3, 4, 5, 6, 8, 10, 12, 24, and 48 hours[d] |

**Table S1**. Description of Phase 1 and 3 TBP-PI-HBr studies included in the population PK analyses

| Study | Phase | Title | Subjects/Patients (n)[a] | TBP-PI-HBr dosing regimens[b] | Scheduled PK sample collection times |
|---|---|---|---|---|---|
| Study SPR994-104 (Study 104, NCT04238195) [3] | 1 | A Single-Dose, Randomized, Placebo- and Active-Control, Four-Way, Cross-Over Study for the Evaluation of the Effect of Tebipenem Pivoxil Hydrobromide (TBPM-PI-HBr) on the QT/QTc Intervals in Adult Healthy Subjects | 24 | 600 or 1200 mg PO single dose | Pre-dose and at 0.5, 0.75, 1, 1.5, 2, 3, 4, 6, 8, 10, 12, and 24 hours |
| Study SPR994-301 (ADAPT-PO, NCT03788967) [4] | 3 | A Phase 3, Randomized, Double-blind, Double-dummy, Multicenter, Prospective Study to Assess the Efficacy, Safety and Pharmacokinetics of Orally Administered Tebipenem Pivoxil Hydrobromide (SPR994) Compared to Intravenous Ertapenem in Patients with Complicated Urinary Tract Infection (cUTI) or Acute Pyelonephritis (AP) | 679[e] | 600 mg PO q8h for 7-10 days[f] | Intense sampling[g]: Day 2 at 0.25, 0.5, 1, 2, and 8 hours<br><br>Sparse sampling: Day 2 at 1, 4, and 8 hours |

Note: h, hours; MAD, multiple ascending dose; mg, milligrams; n, number of subjects/patients; PK, pharmacokinetic; PO, oral; q8h, every 8 hours; SAD, single ascending dose; TBP-PI-HBr, tebipenem pivoxil hydrobromide.

a. Represents the number of subjects/patients considered for the PK population analysis, which included those who received at least one dose of TBP-PI-HBr regardless of formulation and had plasma PK data available. This count by study includes any subjects/patients with outlier samples or samples with concentrations below the limit of quantitation and those with any other missing information that were subsequently evaluated for exclusion.
b. Only TBP-PI-HBr dosing regimens utilizing the IR tablet formulation included. While other formulations were included in the SAD portion of Study 101, only the dosing regimens of the IR tablet are included here (n = 36). The MAD portion of Study 101 and the remaining studies all utilized the IR tablet.
c. Samples also collected on Day 7 at the same times for the food-effect cohorts.
d. Data from Day 5 of Cohort 5 was not utilized in these analyses.
e. 32 patients were excluded due to samples that were below the limit of quantitation or outlier removal that resulted in insufficient PK samples.
f. TBP-PI-HBr dose adjustment for renal impairment allowed.
g. Intense sampling was performed on the first 70 patients enrolled in the study. Samples were collected on Day 1 for subjects enrolled under Protocol Version 1.

**Figure S1.** Semi-log plot of median ($25^{th}$ – $75^{th}$) plasma concentrations versus time, stratified by Phase 1 study based on data from healthy subjects or patients with renal impairment after administration of single dose TBP-PI-HBr 600 mg PO

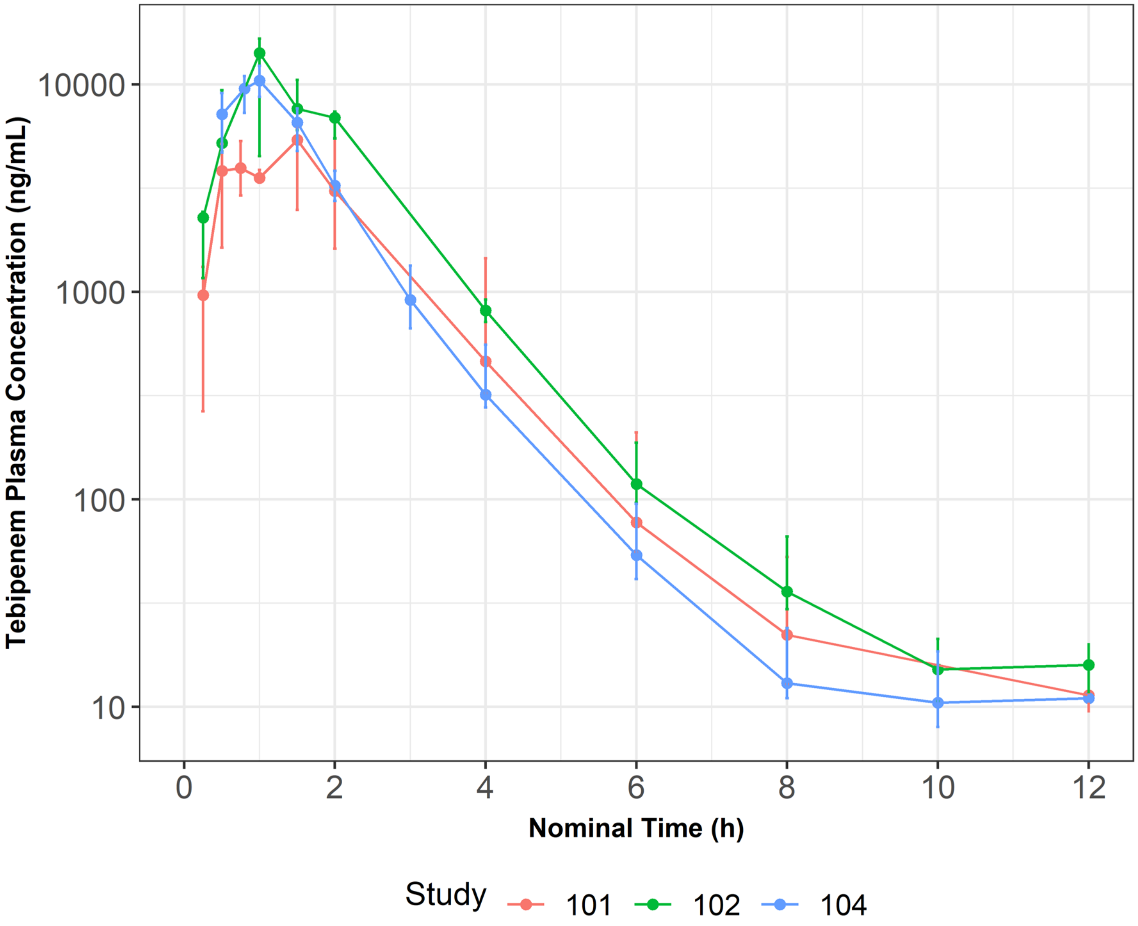


Note: Study 101 data is Cohort 1 only (CLcr ≥ 90 mL/min/1.73 $m^2$).

**Figure S2.** Semi-log scatterplot of observed plasma concentrations versus time based on data for patients from the ADAPT-PO study, colored by dosing regimen and paneled by renal function group

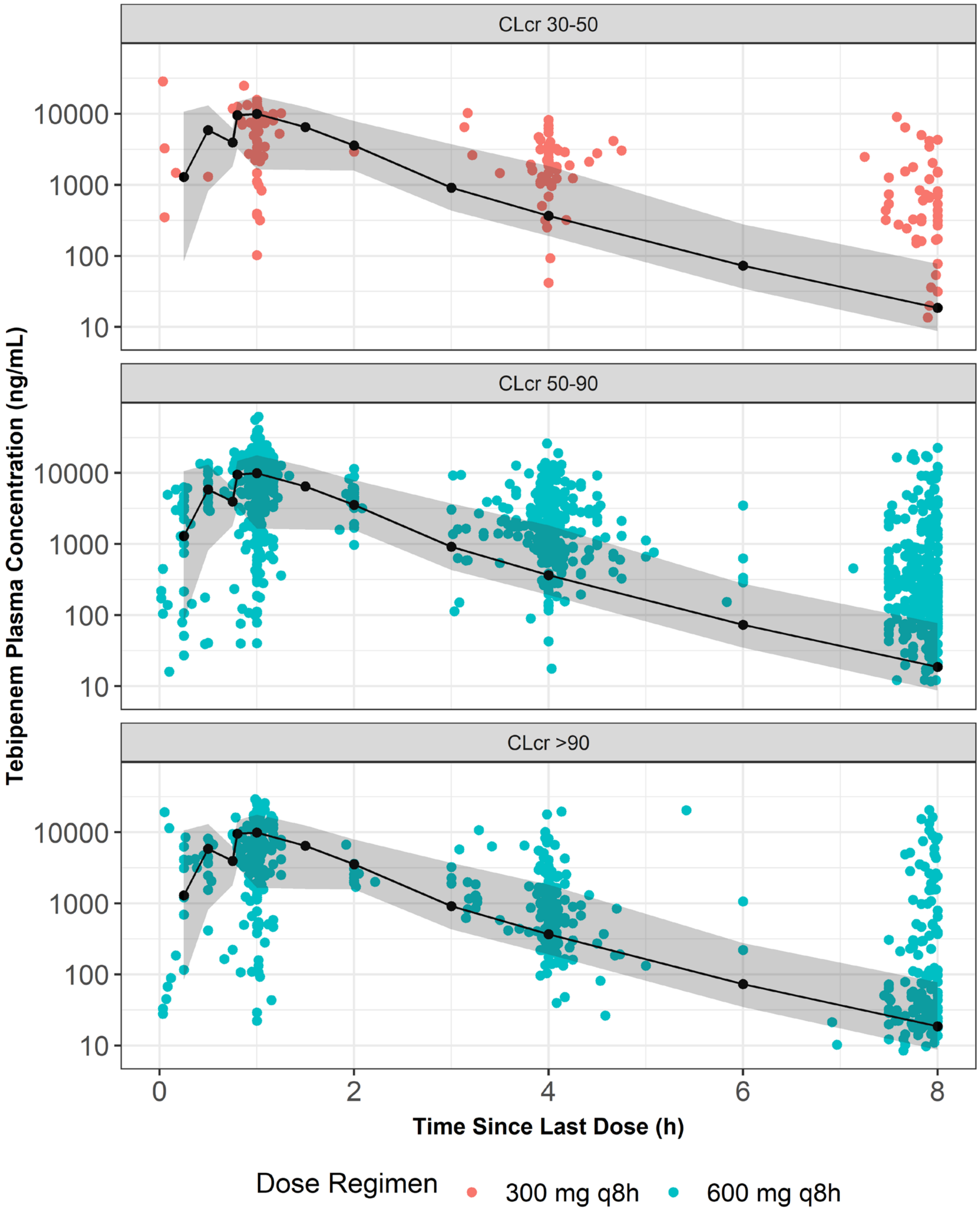


Note: Black line and grey shaded region shown in each panel is median (5th to 95th percentiles) for 600 mg single dose from Phase 1 shown for reference. Note that the 30-50 panel includes patients with CLcr >30 and ≤50 mL/min, and the 50-90 panel includes patients with CLcr >50 and <90 mL/min.

**Figure S3.** Schematic of base structural model applied to the pooled Phase 1 and Phase 3 data

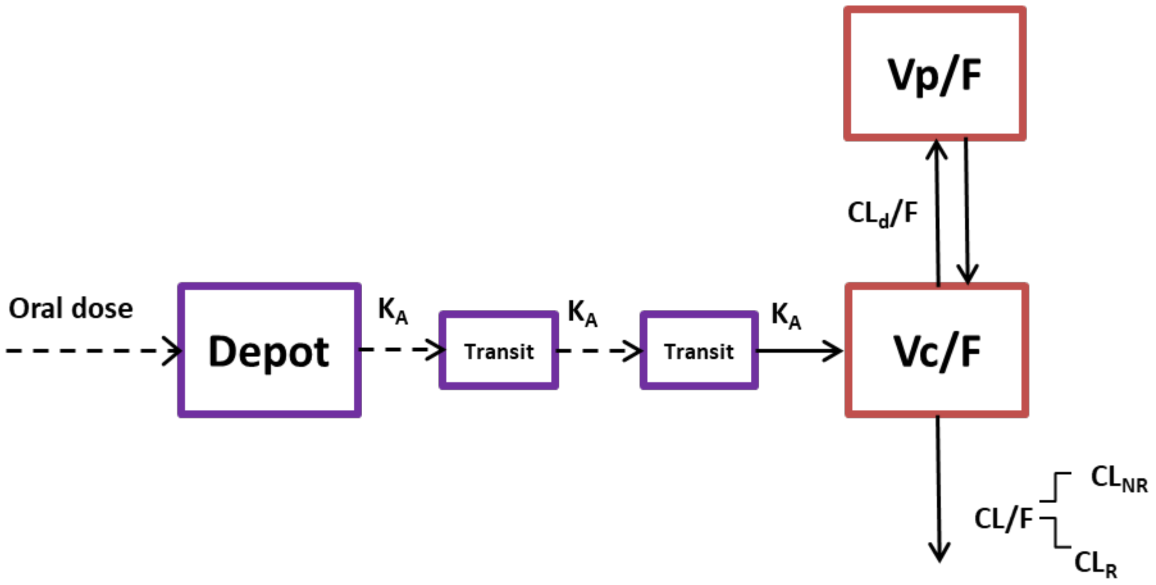

**Figure S4.** Representative plots of individual post-hoc predicted plasma PK profiles overlaid upon observed data for subjects in the Phase 1 studies or patients from the ADAPT-PO study after the first TBP-PI-HBr 600 mg PO dose

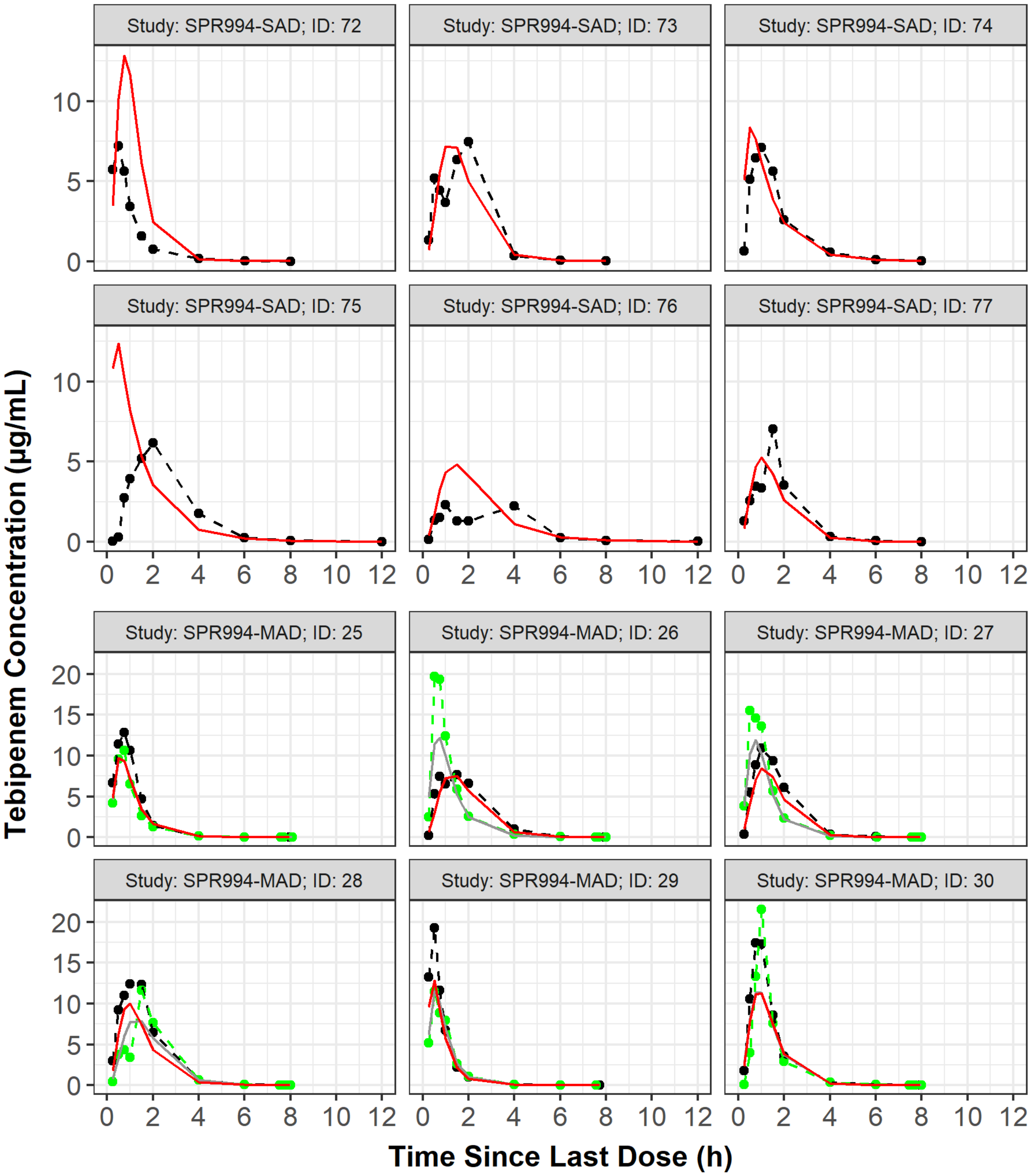

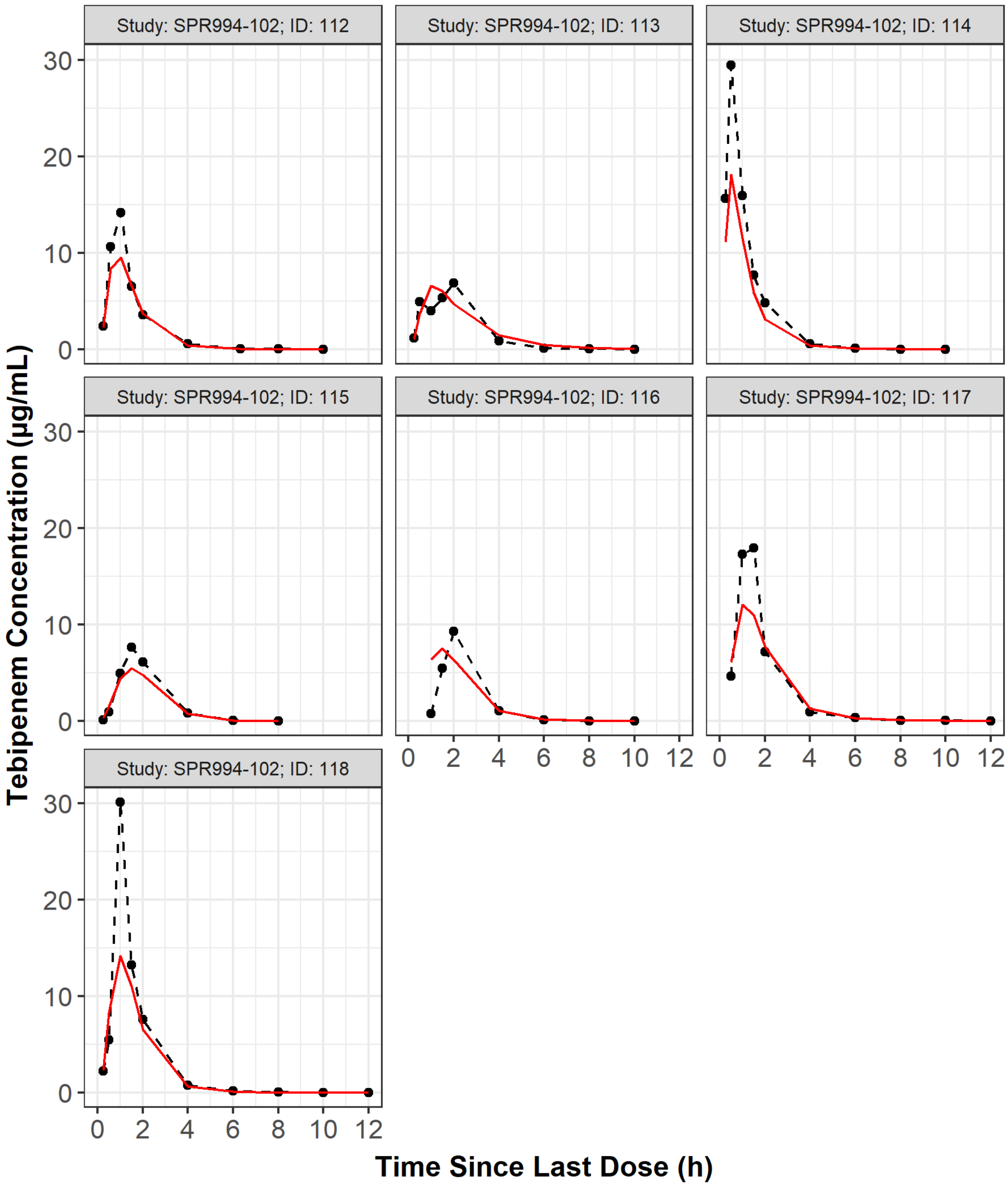
Study: SPR994-102; ID: 112
Study: SPR994-102; ID: 113
Study: SPR994-102; ID: 114
Study: SPR994-102; ID: 115
Study: SPR994-102; ID: 116
Study: SPR994-102; ID: 117
Study: SPR994-102; ID: 118
Tebipenem Concentration (µg/mL)
Time Since Last Dose (h)
0
10
20
30
0
2
4
6
8
10
12

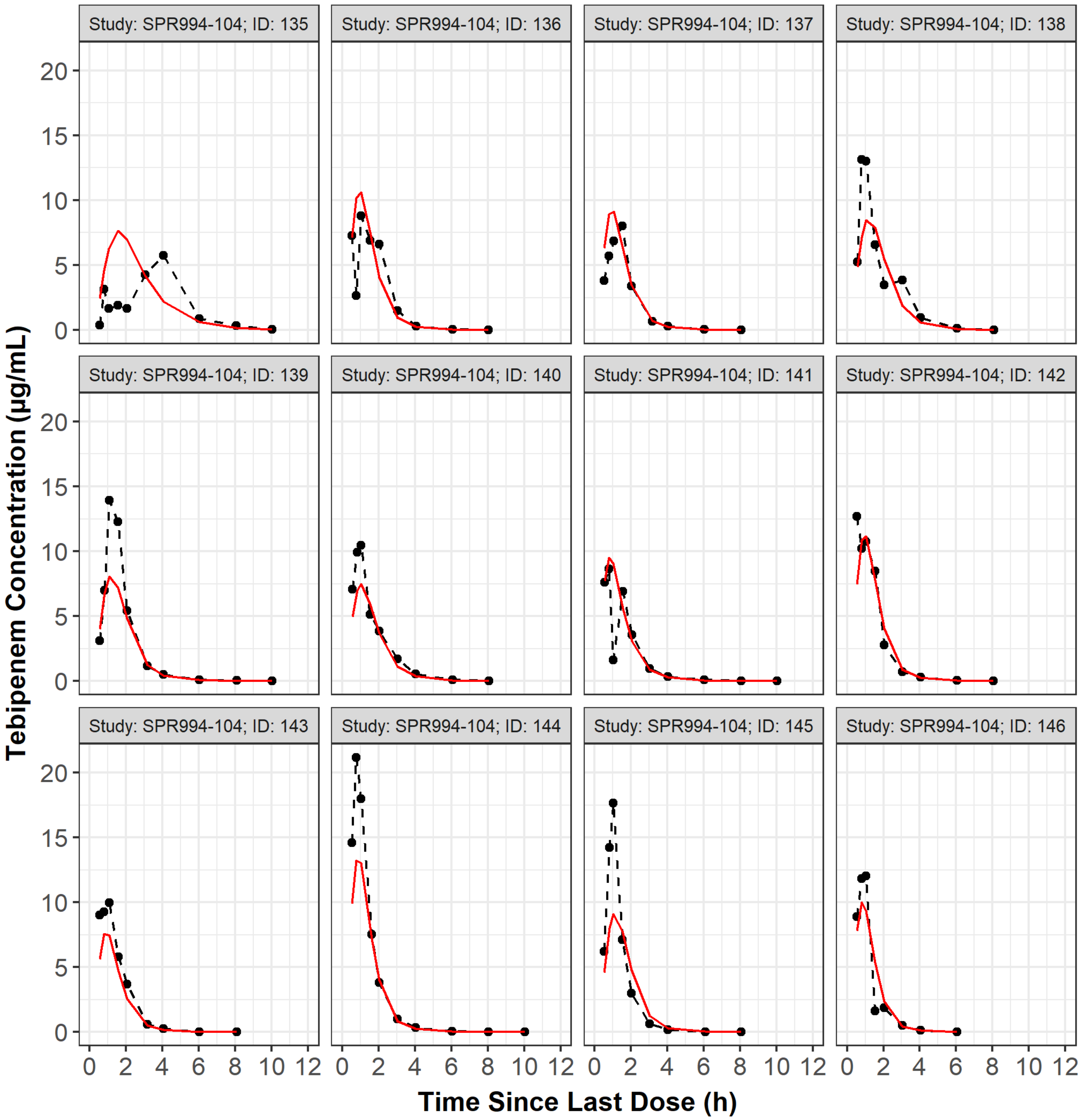
Study: SPR994-104; ID: 135
Study: SPR994-104; ID: 136
Study: SPR994-104; ID: 137
Study: SPR994-104; ID: 138
Study: SPR994-104; ID: 139
Study: SPR994-104; ID: 140
Study: SPR994-104; ID: 141
Study: SPR994-104; ID: 142
Study: SPR994-104; ID: 143
Study: SPR994-104; ID: 144
Study: SPR994-104; ID: 145
Study: SPR994-104; ID: 146
Tebipenem Concentration (µg/mL)
Time Since Last Dose (h)
0
5
10
15
20
0 2 4 6 8 10 12

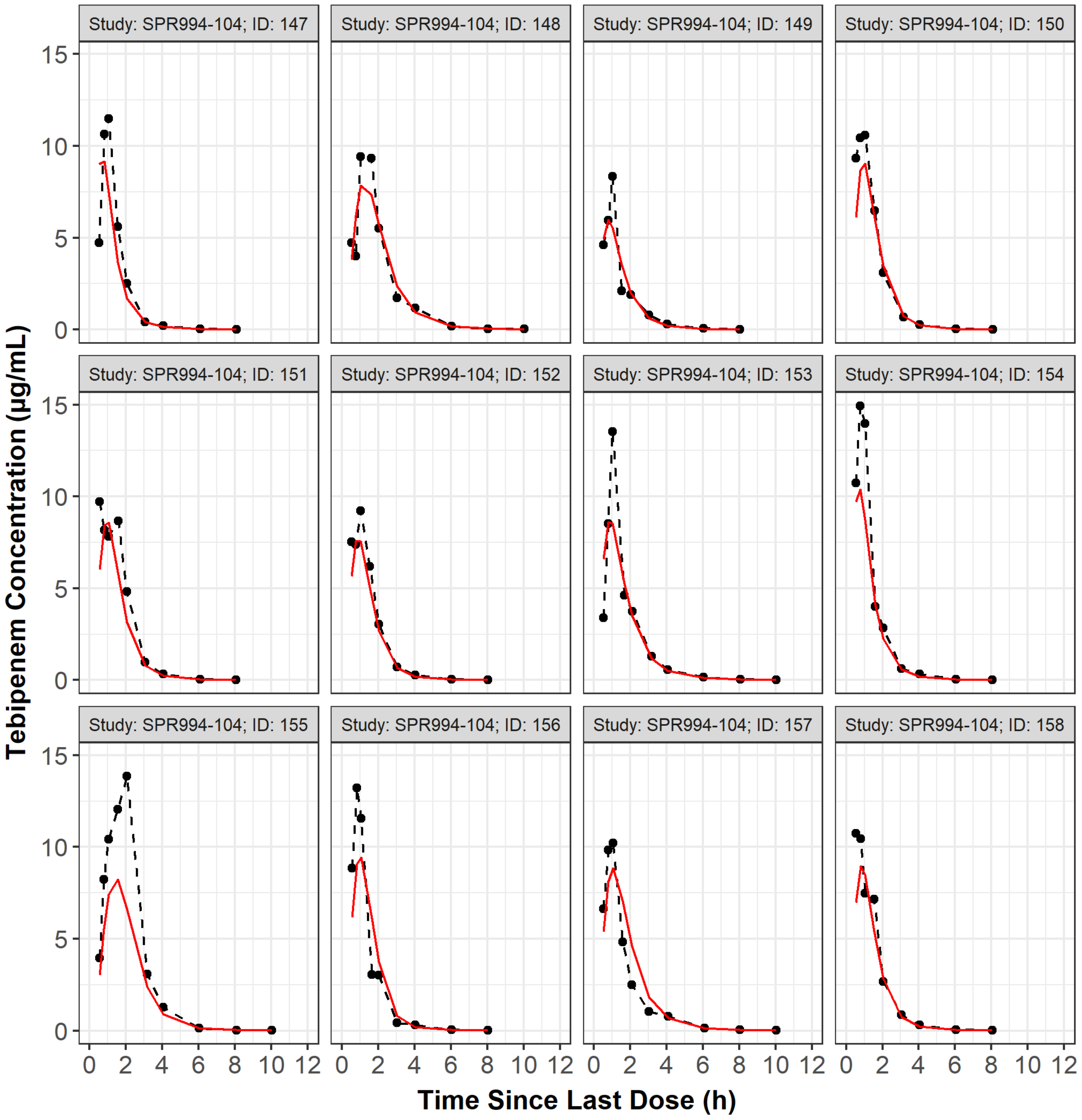
Study: SPR994-104; ID: 147
Study: SPR994-104; ID: 148
Study: SPR994-104; ID: 149
Study: SPR994-104; ID: 150
Study: SPR994-104; ID: 151
Study: SPR994-104; ID: 152
Study: SPR994-104; ID: 153
Study: SPR994-104; ID: 154
Study: SPR994-104; ID: 155
Study: SPR994-104; ID: 156
Study: SPR994-104; ID: 157
Study: SPR994-104; ID: 158
Tebipenem Concentration (µg/mL)
Time Since Last Dose (h)

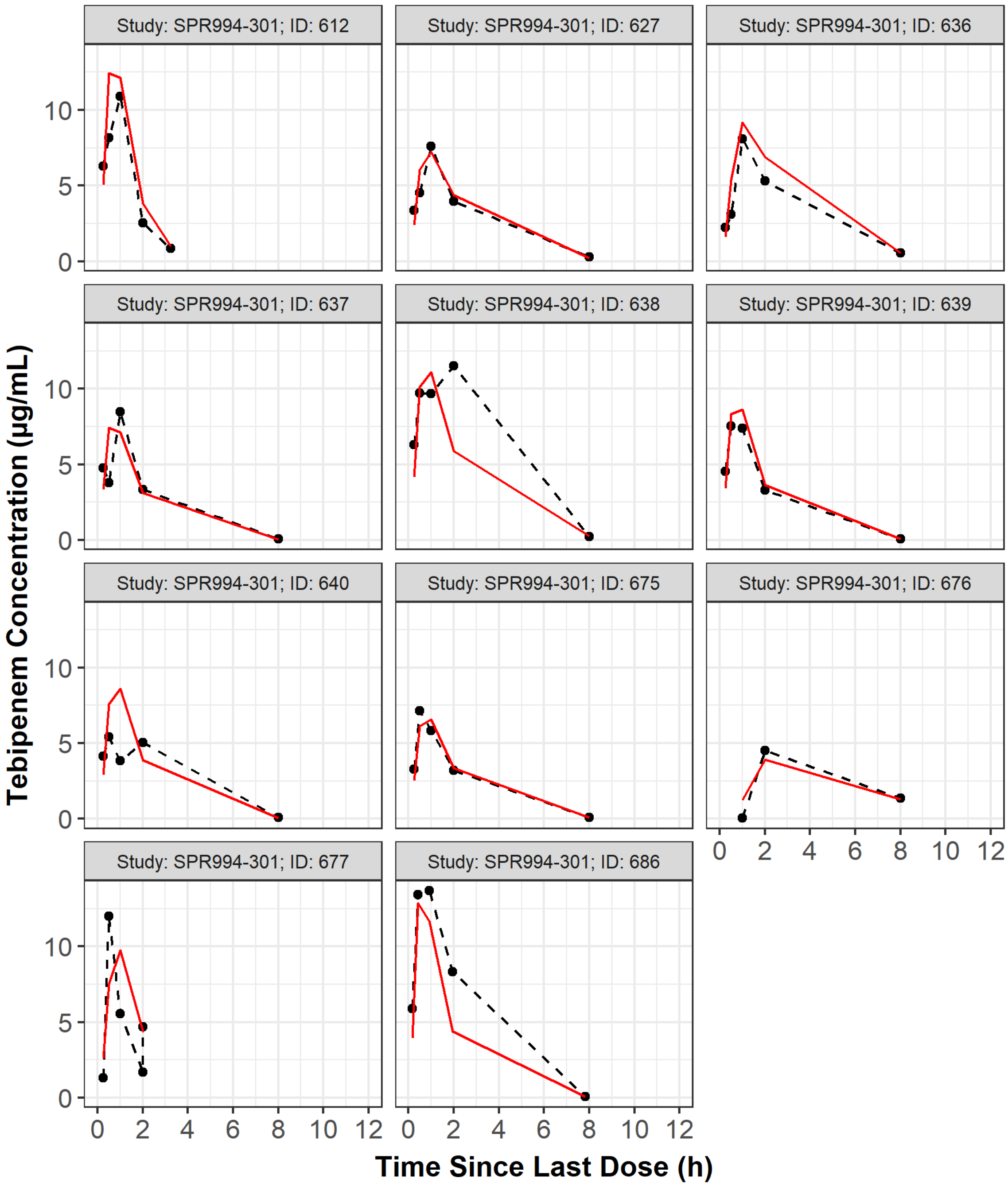


Note: The black dotted lines represent the observed values, and the red solid lines represent the model-predicted values. For the MAD portion of Study 101, the green lines show the observed concentrations obtained with multiple dosing.

**Figure S5.** Standard goodness-of-fit plots for the final population PK model (urine concentrations), colored by dose

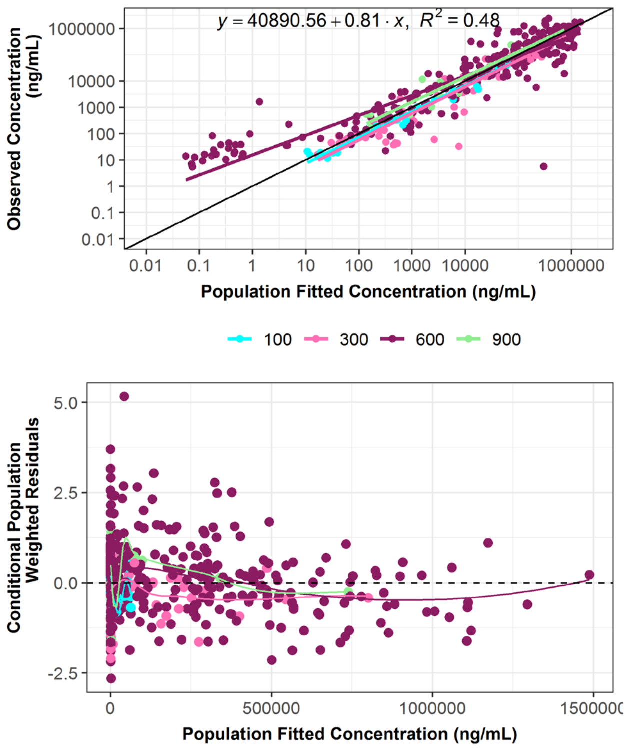


Note: Dose levels are in milligrams (mg).

**Figure S6.** Box-and-whisker plots of tebipenem $AUC_{0\text{-}24}$ on Day 1 in subjects from Study 301, stratified by TBP-PI-HBr dose

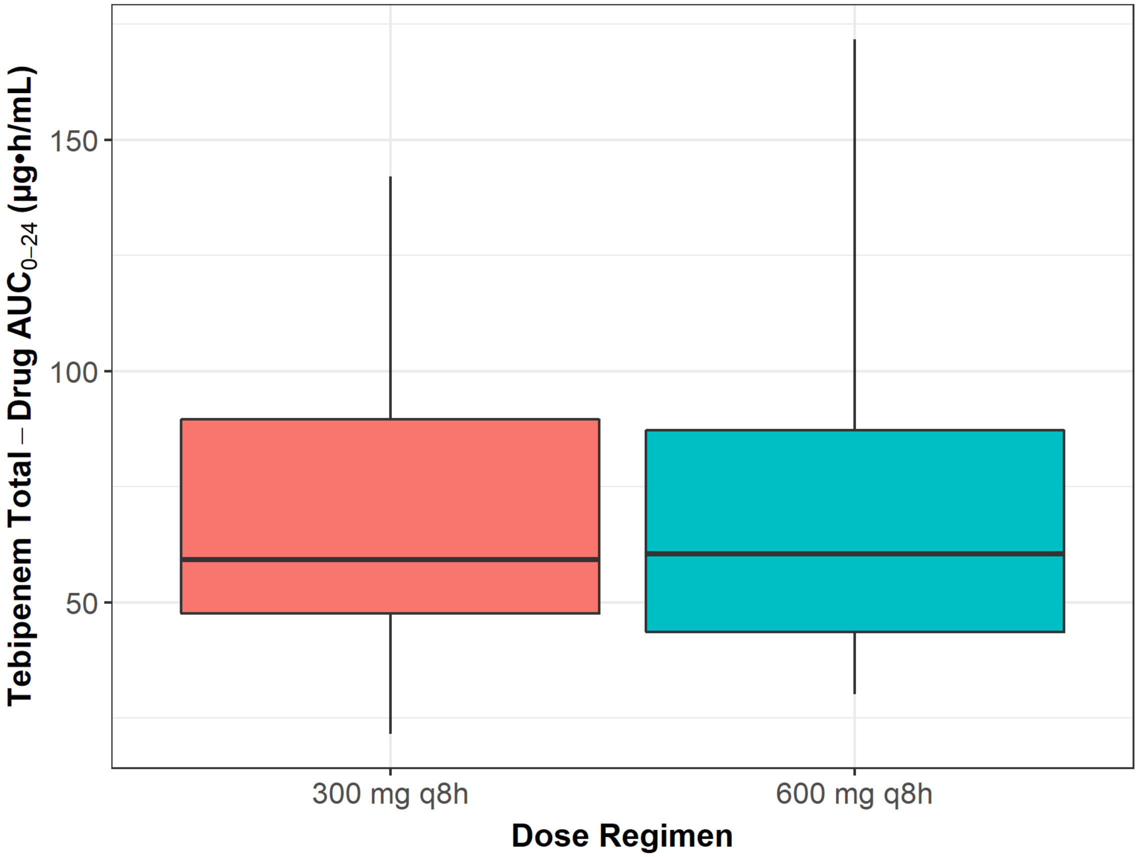


Note: Horizontal line is median, box extends from 25th to 75th percentile, whiskers extend from 5th to 95th percentile. Subjects were randomized to the doses based on baseline CLcr. Patients with baseline CLcr >50 mL/min received TBP-PI-HBr 600 mg q8h while those with baseline CLcr 30-50 mL/min.

## References


1. Eckburg PB, Jain A, Walpole S, et al. Safety, pharmacokinetics, and food effect of tebipenem pivoxil hydrobromide after single and multiple ascending oral doses in healthy adult subjects. Antimicrob Agents Chemother. 2019;63(9):e00618-19.
2. Patel G, Rodvold KA, Gupta VK, Bruss J, Gasink L, Bajraktari F, Lei Y, Jain A, Srivastava P, Talley AK. Pharmacokinetics of oral tebipenem pivoxil hydrobromide in subjects with various degrees of renal impairment. Antimicrob Agents Chemother. 2022;66(5):e0240721.
3. Gupta VK, Maier G, Eckburg P, Morelli L, Lei Y, Jain A, Manyak E, Melnick D. Ranomized, double-blind, placebo- and positive-controlled crossover study of the effects of tebipenem pivoxil hydrobromide on QT/QTc intervals in healthy subjects. Antimicrob Agents Chemother. 2021;65:e00145-21.
4. Eckburg PB, Muir L, Critchley IA, Walpole S, Kwak H, Phelan A-M, Moore G, Jain A, Keutzer T, Dane A, Melnick D, Talley AK. Oral tebipenem pivoxil hydrobromide in complicated urinary tract infection. New Engl J Med. 2022;386(14):1327-1338.
